# Supplementary material for: Transgenic Rice Plants Expressing Artificial miRNA Targeting the Rice Stripe Virus MP Gene Are Highly Resistant to the Virus
Source: Biology (Basel). 2022 Feb 19;11(2):332. doi: 10.3390/biology11020332 (PMC8869529; doi:10.3390/biology11020332)
Supplement: Supplementary file 1 [file biology-11-00332-s001.zip › biology-1537143-supplementary.pdf]

Table S1. Primers used for analysis.

**Primer name**	**Sequence (5'-3')**
Primer-F1	GGATCCCTGTAGCAGCAGCAGTTTCTGAACTACATTAGTCGTCAGGAGATACAGTTTG
Primer-R1	GAGCTCGCCTAGCAGCAGGAATTTCTGAACTACATTAGTCGTAGAGAGGCAAAAGTG
amiR MP	TGCTGATTACATCAAGTCTTT
35S	TCATTTCATTTGGAGAGAACACGGG
NOS	AGACCGGCAACAGGATTCAATC
PCV-F1	TAACTCGACGACAACAGTCGCAGTG
PCV-F2	TAGAGCAGCTTGAGCTTGGATCAG
PCV-F3	ATGAATCACTGGTTGATCGGTGGC
PCV-F4	CAAGAACCAAGGATAACTCGACGAC
PCV-F5	GGCATGGGAGTAGGGACTAAAGGT
LAD2	ACGATGGACTCCAGAGVNVNNHGGTA
LAD3	ACGATGGACTCCAGAGHNVNNNCCAC
LAC1	ACGATGGACTCCAGAG
RT-MP-F	AGCTCAGGGAAGCTCTTGCTGGT
RT-MP-R	CACTAGAGGATAAGGCTATAAACC
RT-UBC-F	TTTCGGTCCTGATGATACTCCC
RT-UBC-R	CACAGAGCAAAGACTGGATTGA
RT-CP-F	CACCGAGGACACTATCCCATAC
RT-CP-R	CAAAGATGCGTTGTCTTACCTG
NB-MP-F1	CAGAACTGTGTCATCTCCAGAACTC
NB-MP-R1	AGCTGGACAAATGGTTTATAGCCTT
NB-MP-F2	AAGCTGTTGGAGGTGCTTTCACT
NB-MP-R2	CTACATGATGACAGAAACTTCAGAT

Table S2. Plant height of homozygous transgenic plants amiR MP-24.

**Transgenic line**	**Plant height（cm） (18 plants)**						Average	*P* value
	84	84	86	**79**	84	83		
amiR MP-24	80	80	82	84	82	84	85.4±2.7	
	89	88	83	87	87	89		
	82	83	88	87	89	84		0.189
WT	87	87	85	84	80	84	84.2±3.0	
	89	89	88	86	83	83		

Table S3. Tiller number of homozygous transgenic plants amiR MP-24.

**Transgenic line**	**Tiller number (18 plants)**						Average	*P* value
	7	6	5	6	5	7		
amiR MP-24	9	6	6	9	6	9	7.1±1.6	
	5	7	10	8	8	9		
	7	9	6	8	8	8		0.649
WT	8	8	9	5	7	9	7.3±1.2	
	7	8	6	6	5	8		

Table S4. seed-setting rate of homozygous transgenic plants amiR MP-24.

**Transgenic line**	**seed-setting rate（%）**							Average	*P* value
amiR MP-24	93.87	94.97	93.12	93.23	93.16			93.67±0.79	0.819
WT	89.81	90.16	95.60	96.26	96.93	94.39	94.71	93.9±2.86	

Table S5 Grains per spike of homozygous transgenic plants amiR MP-24

**Transgenic line**	**Grains per spike（grains）**							Average	*P* value
amiR MP-24	199	189	203	179	177			189.4±11.6	0.378
WT	141	165	174	206	158	215	185	177.7±26.3	
