# Supplementary material for: Transgenic Rice Plants Expressing Artificial miRNA Targeting the Rice Stripe Virus MP Gene Are Highly Resistant to the Virus
Source: Biology (Basel). 2022 Feb 19;11(2):332. doi: 10.3390/biology11020332 (PMC8869529; doi:10.3390/biology11020332)
Supplement: Supplementary file 1 [file biology-11-00332-s001.zip › supplementary Figures.pdf]

Supplementary Figure S1

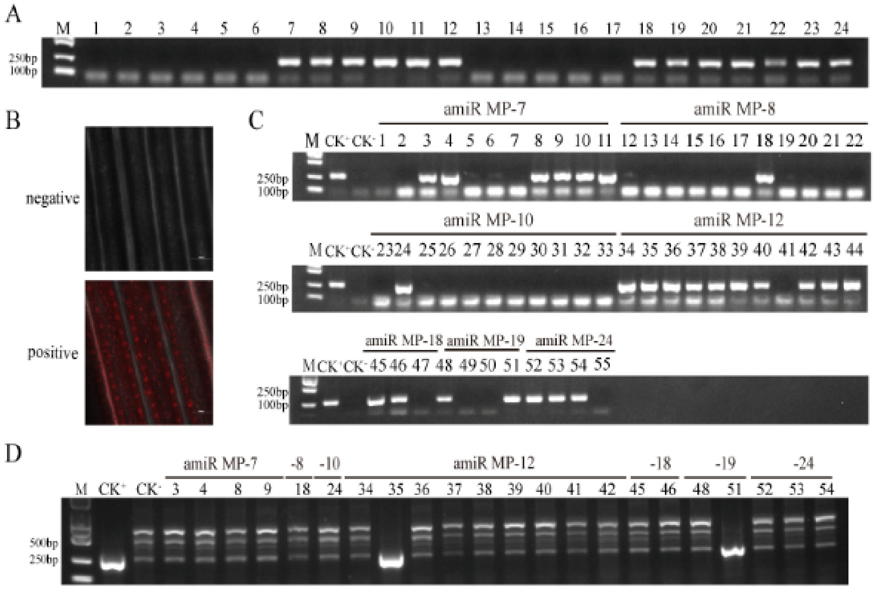

Figure S1 Selection of positive marker-free transgenic plants.

A PCR detection confirming successful transformation of amiR MP in *Oryza sativa* L. var. Zhegeng-88. **B** Confocal microscope observation of red fluorescence (positive) or no red fluorescence (negative) in leaves of T_1 transgenic plants. **C** PCR detection of amiR MP in the RFP-free transgenic plants. *CK+*, the plasmid of 1300 pre-amiR MP was used as positive control for PCR, *CK-*, the DNA of Wild type (Zhegeng-88) plants was used as the negative control. No bands were obtained from the wild type plants, demonstrating the specificity of the primers. **D** PCR detection of marker gene RFP to confirm that the positive amiR MP transformed plants were without the selective marker.
